# Prognostic value of the pretreatment pan-immune-inflammation value in patients with head and neck squamous cell carcinoma: a systematic review and meta-analysis

**DOI:** 10.3389/fonc.2026.1743144

**Published:** 2026-03-10

**Authors:** Te Li, Genping Li

**Affiliations:** 1 Department of Otolaryngology, The Seventh People’s Hospital of Chongqing, Chongqing, China; 2 Department of Otolaryngology, The People’s Hospital of Chongqing Liang Jiang New Area, The Affiliated Liang Jiang Hospital of Chongqing Medical University, Chongqing, China

**Keywords:** head and neck squamous cell carcinoma, pan-immune-inflammation value, overall survival, disease-free survival, distal metastasis-free survival, local recurrence-free survival

## Abstract

**Background:**

The prognostic significance of the pan-immune-inflammation value (PIV) in head and neck squamous cell carcinoma has been comprehensively documented. Nevertheless, its exact role remains ambiguous. The objective of this study is to perform a systematic exploration of the correlation between the pretreatment PIV and survival outcomes in this population.

**Methods:**

An extensive and systematic search of the literature was conducted through electronic databases, including Web of Science, PubMed, and Embase. The search period covered from inception to October 1, 2025. The primary endpoint was survival outcomes. Hazard ratios (HRs) and their 95% confidence intervals (CIs) for survival outcomes were retrieved. A random-effects model was employed to integrate the pooled findings. This meta-analysis was prospectively registered with PROSPERO (CRD420251170558).

**Results:**

A total of twelve studies, encompassing 5,056 patients, were included. The pooled results revealed that patients in the high PIV group exhibited significantly inferior overall survival (12 studies; HR = 2.62; 95% CI: 2.00 – 3.44; I² = 74%) and disease-free survival (9 studies; HR = 2.34; 95% CI: 1.69 - 3.26; I² = 79%) when compared to those in the low PIV group. Subgroup analyses buttressed the prognostic significance of PIV for overall survival and disease-free survival across diverse geographical regions, tumor stages, and treatment strategies. Moreover, evidence aggregated from limited studies indicated that a higher PIV was associated with a worse distal metastasis-free survival (3 studies; HR = 2.04; 95% CI: 1.13 - 3.67; P = 0.02; I² = 94%) and a marginally poorer local recurrence-free survival (3 studies; HR = 1.20; 95% CI: 1.00 - 1.44; P = 0.05; I² = 0%).

**Conclusions:**

Our findings indicate that the pretreatment PIV has the potential to serve as a valuable biomarker for predicting the survival outcomes of patients with head and neck squamous cell carcinoma.

**Systematic review registration:**

https://www.crd.york.ac.uk/prospero/, identifier CRD420251170558.

## 1 Background

Head and neck squamous cell carcinoma (HNSCC), ranking as the sixth most prevalent cancer globally, constitutes a heterogeneous group of tumors that originate from multiple anatomical sites, such as the oral cavity, oropharynx, hypopharynx, and larynx (1). Conventionally, the management of these tumors has been highly dependent on TNM staging and histologic grading (2). However, relying solely on these tumor-derived parameters still presents a significant challenge in effectively guiding treatment selection and prognosis evaluation for head and neck surgeons (2). Consequently, there is an urgent necessity to identify reliable biomarkers that can more accurately predict both prognosis and treatment response (3).

A substantial body of evidence highlights the crucial role of the host’s inflammatory and immune states in regulating the progression, treatment response, and survival patterns of cancer patients (4, 5). Based on this understanding, several inflammation/immune-associated biomarkers have come to light for predicting clinical outcomes in oncology. These include the monocyte-to-lymphocyte ratio (MLR) (6), neutrophil-to-lymphocyte ratio (NLR) (7), and platelet-to-lymphocyte ratio (PLR) (8). Lately, a novel prognostic biomarker known as the pan - immune - inflammation value (PIV) has drawn the attention of clinicians across the globe (9–11). PIV was first presented by Fuca et al. (12) in 2020 as a prognostic index for metastatic colorectal cancer patients receiving chemotherapy in combination with targeted therapy. PIV combines neutrophils, platelets, monocytes, and lymphocytes into one metric and is calculated as follows: serum neutrophil × platelet × monocyte ÷ lymphocyte. In comparison to simpler counterparts like NLR, MLR, and PLR, it has shown better prognostic accuracy (9). A recent meta-analysis encompassing 30 studies has established that elevated PIV levels are correlated with inferior overall survival (OS) and progression-free survival (PFS) in patients with solid tumors (9). Correspondingly, in the context of hematologic malignancies, Ucar et al. (13) further identified PIV as an independent prognostic factor for OS. Nevertheless, the precise implications for the prognosis of patients with HNSCC have yet to be fully elucidated.

As far as we know, no systematic review and meta-analysis has been conducted to explore the prognostic significance of the PIV in patients with HNSCC. In this study, our aim is to comprehensively summarize the existing evidence and clarify the prognostic value of the pretreatment PIV in HNSCC patients.

## 2 Methods

### 2.1 Search strategy

The present systematic review and meta-analysis was performed in line with the Preferred Reporting Items for Systematic Reviews and Meta-Analyses (PRISMA) guidelines (14) and was prospectively registered with PROSPERO (CRD420251170558). Relevant studies from Web of Science, PubMed, and Embase were systematically examined from the establishment of these databases to October 1, 2025. A comprehensive search strategy employing a combination of keywords was utilized to retrieve pertinent studies: (PIV OR pan-immune-inflammation value) AND (head and neck cancer OR oropharyngeal cancer OR laryngeal cancer OR salivary gland cancer OR hypopharyngeal cancer OR nasopharyngeal cancer OR oral cavity cancer OR paranasal sinus cancer OR nasal cavity cancer). Language restrictions were not imposed during the search process. In addition, Google Scholar and the references of included articles were manually searched for gray literature.

### 2.2 Study selection

The inclusion criteria were established using the PICOS approach (15) as follows: P (Population): Patients diagnosed as HNSCC; I (Intervention): High PIV; C (Comparator): Low PIV; O (Outcome): Survival outcomes; S (Study design): Randomized controlled trials and cohort studies.

Exclusion criteria: (1) Studies presented as letters, case reports, or abstracts; (2) PIV was not presented as a binary variable; (3) Duplicate data.

### 2.3 Data extraction and quality assessment

Data extraction was independently carried out by two authors, and all the collected information was cross-validated. The data extracted covered various details, including the first author, publication year, country, study duration, study design, sample size, blood sampling time, cut-off method, cut-off value of PIV, age, sex, cancer site, tumor stage, primary treatment modality, median follow-up time, and survival outcomes. In this study, the survival outcomes included OS, PFS, disease-free survival (DFS), local recurrence-free survival (LRFS) and distal metastasis-free survival (DMFS). It is worth noting that, in this meta-analysis, as PFS and DFS have similar endpoints, they were analyzed together as one outcome measure - DFS - following previous reports (16–18).

The quality of the included studies was appraised by applying the Newcastle-Ottawa Scale (NOS) (19). This scale is composed of eight pre-established criteria. Following a comprehensive assessment, each individual study was given a final score within the range of 0 to 9. Studies with scores from 7 to 9 were regarded as representing high-quality research.

### 2.4 Statistical analysis

For these survival outcomes, hazard ratios (HRs) together with their 95% confidence intervals (CIs) were utilized as the effect size. When survival data were not directly presented in the literature, we retrieved them from the survival curves by applying the methods detailed by Tierney et al. (20). The I^2^ statistics were used to evaluate the statistical heterogeneity among the included studies. Considering the heterogeneity of the clinical backgrounds of the included studies, a random-effects model was adopted for all meta-analyses. Additionally, subgroup analysis and sensitivity analysis were carried out to explore the source of heterogeneity and to investigate the stability of the pooled outcomes. The funnel plot, along with Egger’s test and Begg’s test, was used to assess potential publication bias. If the pooled outcomes showed significant publication bias (P<0.10 for either Egger’s test or Begg’s test), trim-and-fill analysis would be implemented. A two-tailed P value <0.05 was considered statistically significant. All statistical analyses were performed using R software, version 4.2.1.

## 3 Results

### 3.1 Study characteristics

**Figure 1:**
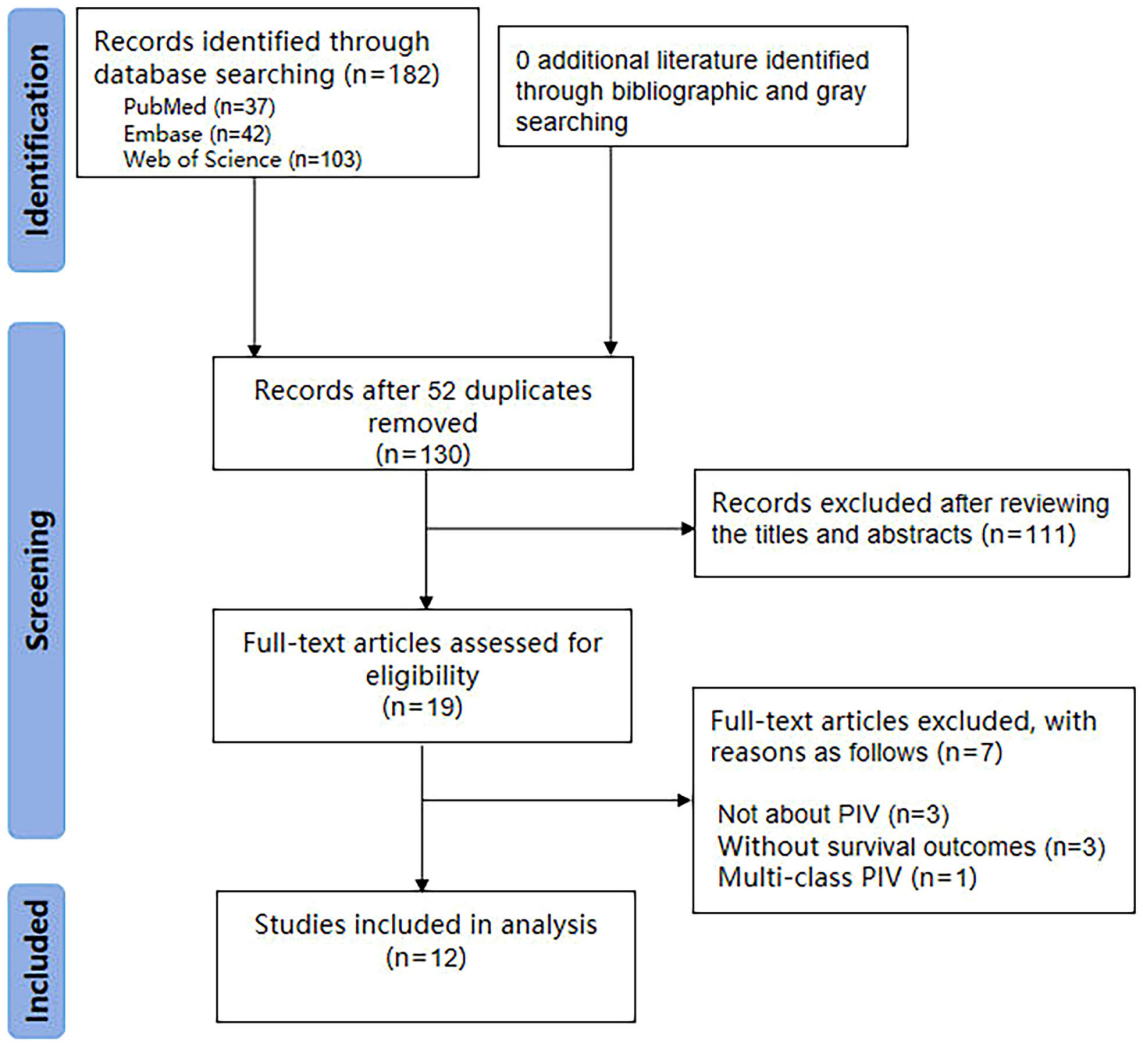
The PRISMA flowchart of study selection.

The databases were searched comprehensively, yielding a total of 182 articles. Duplicates were removed using EndNote X9 software, leaving 130 articles. Following initial title and abstract screening, 19 studies were included. After a careful assessment of full text, 12 studies (21–32) were ultimately included for systematic review and meta-analysis (**Figure 1**). As presented in **Tables 1** and **2**, these studies were published between 2022 and 2025, originating from China (75%) and Turkey (25%), respectively. All of the studies adopted a retrospective research design. These 12 studies involved a total of 13 cohorts comprising 5,056 HNSCC patients. The sample sizes varied from 50 to 860 individuals. Concerning the primary treatment methods used in these studies, surgery was carried out in four (30.7%) cohorts, radiotherapy or chemoradiotherapy was applied in 8 (61.5%) cohorts, and immune checkpoint inhibitors were used in one (7.7%) cohort. The median follow - up time of the included studies ranged from 16.3 to 85.7 months. Notably, these studies showed good quality, with scores ranging from six (2 cohorts [15.4%]) to seven (11 cohorts [84.6%]) (**Supplementary Table S1**).

**Table 1:** Basic information of included studies.

References	Country	Study design	Study interval	Sample size	Sampling time	Cut-off selection method	Cut-off value	Age, years (Median)	Sex (M: F)	Cancer type
Guven, 2022 (21)	Turkey	Retrospective	2005-2020	199	Before treatment	ROC	404	59	180/19	HNSCC
Lai, 2023 (25)	China	Retrospective	2015-2018	441	Within one week	ROC	363.47	49	324/117	NPC
Lien, 2023 (26)	China	Retrospective	2018-2022	192	Before treatment	Median	966	58	176/16	HNSCC
Yeh, 2023 (22)	China	Retrospective	2005-2017	853	4.9 ± 2.8 days	ROC	268	53.5	780/73	OSCC
Koca, 2024 (24)	Turkey	Retrospective	2014-2022	101	Within one month	ROC	478.3	NA	93/8	LC
Shi, 2024 (32)	China	Retrospective	2020-2022	545	Within one week	MSR	277.06	62	465/80	L/PC
Shu, 2024 (27)	China	Retrospective	2014-2019	319	Before treatment	ROC	428	NA	224/95	NPC
Topkan, 2024 (28)	Turkey	Retrospective	2010-2019	179	Within one week	ROC	512	59	140/39	NPC
Zhang, 2024 (29)	China	Retrospective	2014-2019	377	Before treatment	ROC	146.24	49	266/111	NPC
Chen, 2025 (23)	China	Retrospective	2008-2018	161	Within one week	MSR	123.3	NA	151/10	HNSCC
Chen, 2025 (23)	China	Retrospective	2008-2017	50	Within one week	MSR	123.3	NA	45/5	HNSCC
Huang, 2025 (30)	China	Retrospective	2015-2022	779	Within one week	X-tile	180.9	61	474/305	OSCC
Li, 2025 (31)	China	Retrospective	2010-2014	860	Within one week	MSR	244.4	45	221/639	NPC

M, male; F, female; ROC, receiver operator characteristic; MSR, maximally selected rank; HNSCC, head and neck squamous cell carcinoma; NPC, nasopharyngeal carcinoma; OSCC, oral squamous cell carcinoma; LC, laryngeal carcinoma; L/PC, laryngeal/pharyngeal carcinoma NA, not available.

**Table 2:** Survival information of included studies.

References	Tumor stage	Primary treatment	Median follow-up time (Months)	Survival outcomes	Multivariate analysis	NOS
Guven, 2022 (21)	Nonmetastatic	RT/CRT	71.6	OS; DFS	Yes; Yes	7
Lai, 2023 (25)	Mixed	RT/CRT	71	OS	Yes	7
Lien, 2023 (26)	Metastatic	ICI	16.3	OS; PFS	Yes; Yes	6
Yeh, 2023 (22)	Mixed	Surgery	NA	OS; DFS; LRFS; DMFS	Yes; Yes; Yes; Yes	7
Koca, 2024 (24)	Mixed	RT/CRT	23	OS; PFS; LRFS	Yes; Yes; No	7
Shi, 2024 (32)	Nonmetastatic	Surgery	NA	OS; DFS	Yes; Yes	6
Shu, 2024 (27)	Nonmetastatic	RT/CRT	40.4	OS; PFS	Yes; Yes	7
Topkan, 2024 (28)	Nonmetastatic	RT/CRT	85.7	OS; DMFS	Yes; Yes	7
Zhang, 2024 (29)	Mixed	RT/CRT	55.5	OS; PFS; LRFS; DMFS	Yes; Yes; No; Yes	7
Chen, 2025 (23)	Mixed	Surgery	60	OS; DFS	Yes; Yes	7
Chen, 2025 (23)	Mixed	RT/CRT	NA	OS; PFS	Yes; Yes	7
Huang, 2025 (30)	Mixed	Surgery	34.1	OS	Yes	7
Li, 2025 (31)	Nonmetastatic	RT/CRT	NA	OS	Yes	7

RT/CRT, radiotherapy or chemoradiotherapy; ICI, immune checkpoint inhibitor; OS, overall survival; DFS, disease-free survival; PFS, progression-free survival; LRFS, local recurrence-free survival; DMFS, distal metastasis-free survival; NOS, Newcastle-Ottawa Scale; NA, not available.

### 3.2 Relationship between the pretreatment PIV and OS

**Figure 2:**
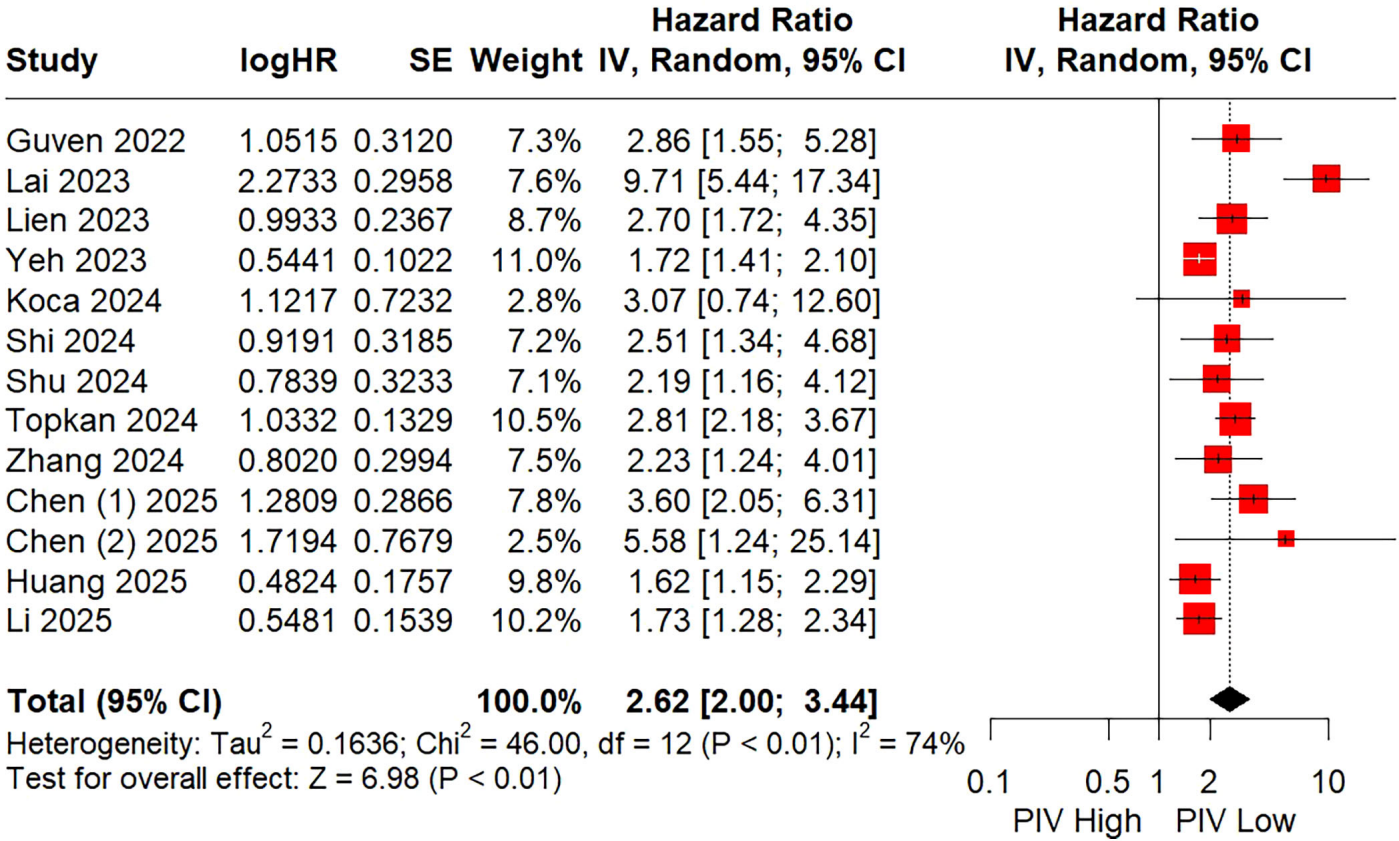
Forest plot assessing the relationship between the pretreatment PIV and overall survival.

All of the included studies with a total of 5,056 individuals explored the prognostic significance of PIV for predicting OS using multivariate COX regression models in HNSCC patients. As shown in **Figure 2** and **Table 3**, the random-effects meta-analysis demonstrated that a higher PIV was significantly linked to poorer OS (HR = 2.62; 95% CI: 2.00 – 3.44), with significant heterogeneity observed (I^2^ = 74%).

**Table 3:** Subgroup analyses of overall survival between the High and Low PIV groups.

Variables	Subgroups	Cohorts, n	Sample sizes, n	HR	95% CI	I^2^ (%)
Total		13	5056	2.62	2.00-3.44	74
Country						
	China	10	4577	2.60	1.84-3.67	78
	Turkey	3	479	2.82	2.23-3.58	0
Sample size						
	>300	7	4174	2.40	1.54-3.75	82
	<300	6	882	2.92	2.40-3.55	0
Sampling time						
	Within one week	6	2836	3.09	1.72-5.55	86
	Others	7	2220	2.32	1.86-2.91	44
Cut-off method						
	ROC	7	2469	2.89	1.86-4.50	83
	MSR	4	1616	2.54	1.62-3.96	57
	Others	2	971	2.04	1.24-3.36	67
Cut-off value						
	>300	6	1431	3.35	2.14-5.26	71
	<300	7	3625	1.97	1.62-2.40	40
Cancer site						
	HNSCC	4	602	3.07	2.27-4.15	0
	NPC	5	2176	2.92	1.64-5.22	86
	OSCC	2	1632	1.70	1.43-2.02	0
	L/PC	2	646	2.59	1.46-4.59	0
Tumor stage						
	Nonmetastatic	5	2102	2.33	1.81-3.00	36
	Mixed	7	2762	3.01	1.76-5.13	84
	Metastatic	1	192	2.70	1.72-4.35	–
Primary treatment						
	Surgery	4	2338	2.08	1.48-2.90	59
	RT/CRT	8	2526	3.01	1.98-4.59	76
	ICI	1	192	2.70	1.72-4.35	–

ROC, receiver operator characteristic; MSR, maximally selected rank; HNSCC, head and neck squamous cell carcinoma; NPC, nasopharyngeal carcinoma; OSCC, oral squamous cell carcinoma; L/PC, laryngeal/pharyngeal carcinoma; RT/CRT, radiotherapy or chemoradiotherapy; ICI, immune checkpoint inhibitor.

Furthermore, subgroup analyses were carried out based on various factors, including country, sample size, sampling time, cut-off method, cut-off value, cancer site, tumor stage, and primary treatment, aiming to investigate the source of heterogeneity and the stability of the pooled outcome in different populations. As shown in **Table 3** and **Supplementary Figure S1**, the combined results of all subgroup analyses showed that patients in the high PIV group had a significantly shorter OS compared to those in the low PIV group (all P values < 0.05). However, none of these parameters contributed to the source of heterogeneity, as certain heterogeneities still presented across each of those subgroup patients. As shown in **Supplementary Figure S3A**, the leave-one-out sensitivity analysis indicated that the study by Lai et al. (25) was the source of heterogeneity (I^2^ decreased to 46%) and the combined outcomes remained stable after omitting each included study (**Supplementary Figure S3A**).

### 3.3 Relationship between the pretreatment PIV and DFS

**Figure 3:**
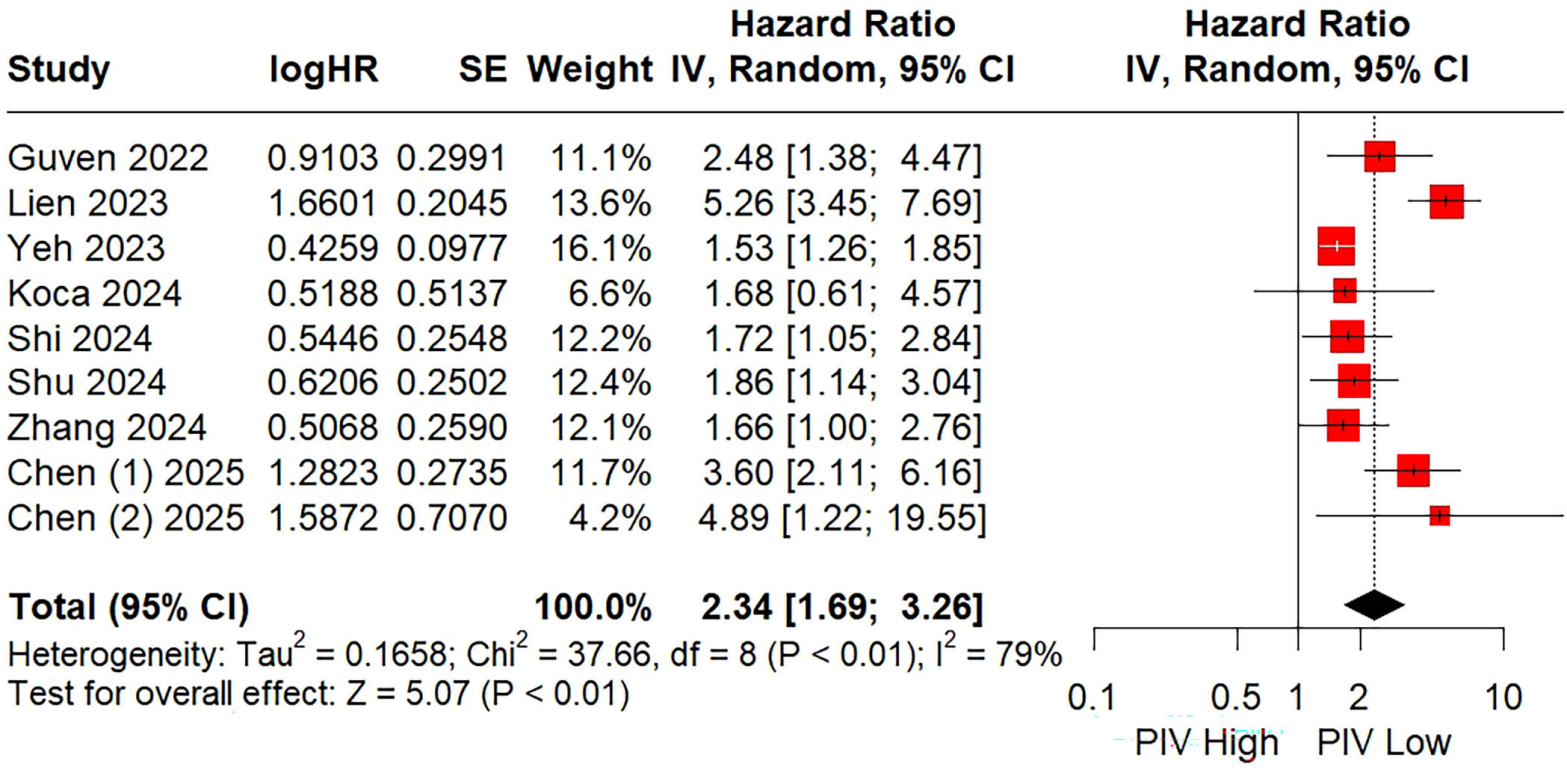
Forest plot assessing the relationship between the pretreatment PIV and disease-free survival.

A total of nine studies, which included 2,797 patients in aggregate, explored DFS with multivariate COX regression models applied. The pooled HR using random-effects model was 2.34 (95% CI: 1.69 - 3.26), indicating that patients in the high PIV group had a worse DFS than patients in the low PIV group (**Figure 3**, **Table 4**), with significant heterogeneity detected (I^2^ = 79%).

**Table 4:** Subgroup analyses of disease-free survival between the High and Low PIV groups.

Variables	Subgroups	Cohorts, n	Sample sizes, n	HR	95% CI	I^2^ (%)
Total		9	2797	2.34	1.69-3.26	79
Country						
	China	7	2497	2.40	1.61-3.59	84
	Turkey	2	300	2.25	1.36-3.74	0
Sample size						
	>300	4	2094	1.59	1.36-1.87	0
	<300	5	703	3.48	2.33-5.20	45
Sampling time						
	Within one week	3	756	2.74	1.48-5.08	58
	Others	6	2041	2.20	1.45-3.34	84
Cut-off method						
	ROC	5	1849	1.64	1.39-1.93	0
	MSR	3	756	2.74	1.48-5.08	58
	Others	1	192	5.26	3.45-7.69	–
Cut-off value						
	>300	4	811	2.70	1.56-4.67	77
	<300	5	1986	2.03	1.40-2.93	63
Cancer site						
	HNSCC	4	602	3.86	2.61-5.70	34
	NPC	2	696	1.76	1.24-2.51	0
	OSCC	1	853	1.53	1.26-1.85	–
	L/PC	2	646	1.72	1.10-2.68	0
Tumor stage						
	Nonmetastatic	3	1063	1.95	1.45-2.64	0
	Mixed	5	1542	2.09	1.38-3.16	63
	Metastatic	1	192	5.26	3.45-7.69	–
Primary treatment						
	Surgery	3	1559	2.03	1.23-3.37	77
	RT/CRT	5	1046	1.98	1.49-2.63	0
	ICI	1	192	5.26	3.45-7.69	–

ROC, receiver operator characteristic; MSR, maximally selected rank; HNSCC, head and neck squamous cell carcinoma; NPC, nasopharyngeal carcinoma; OSCC, oral squamous cell carcinoma; L/PC, laryngeal/pharyngeal carcinoma; RT/CRT, radiotherapy or chemoradiotherapy; ICI, immune checkpoint inhibitor.

Furthermore, subgroup analyses were carried out based on the variables mentioned above. As presented in **Table 4** and **Supplementary Figure S2**, the pooled findings from all subgroup analyses consistently showed that patients in the high PIV group had significantly poorer DFS than those in the low PIV group (all P values < 0.05). Meanwhile, we identified that sample size and cancer site were key variables contributing to the observed heterogeneity. Additionally, sensitivity analysis indicated that the study by Lien et al. (26) led to the significant heterogeneity (I^2^ decreased to 44%) and the prognostic value of PIV was consistent upon sequential exclusion of individual studies (**Supplementary Figure S3B**).

### 3.4 Relationship between the pretreatment PIV and LRFS

**Figure 4:**
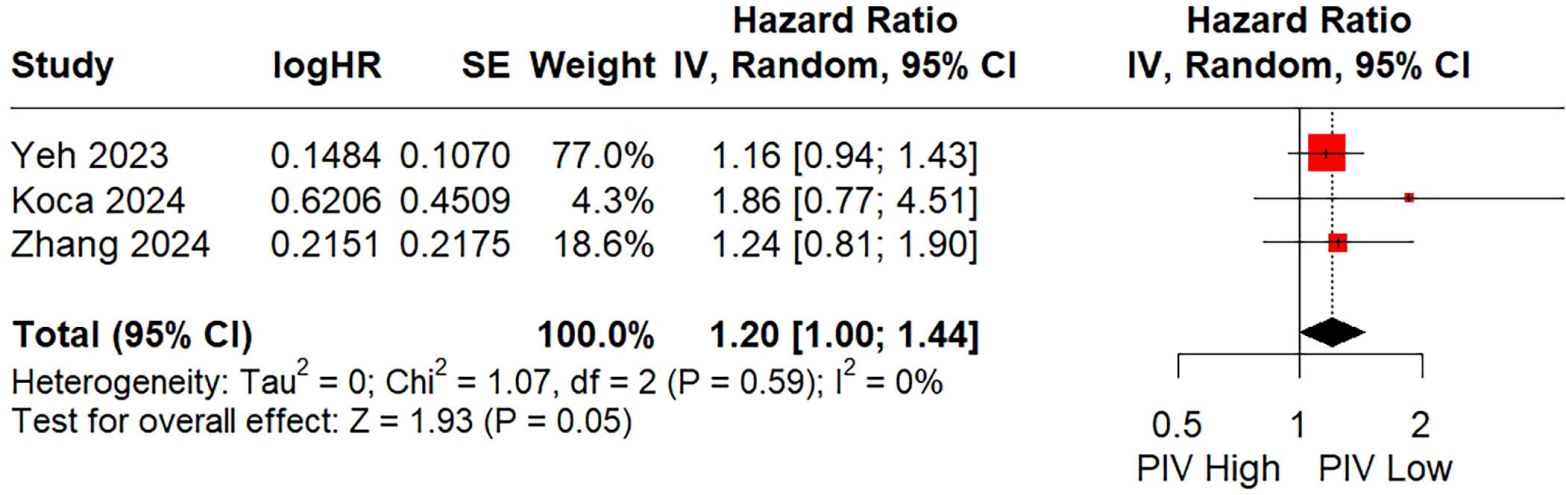
Forest plot assessing the relationship between the pretreatment PIV and local recurrence-free survival.

Three studies involving 1,331 patients explored the relationship between the PIV and LRFS (two employed univariate analysis and one applied multivariate analysis). The random-effects combined result indicated that a higher PIV was slightly linked to a worse LRFS (HR = 1.20; 95% CI: 1.00 - 1.44; P = 0.05; I² = 0%; **Figure 4**). Given the limited number of included references and the consistent negative findings across the included studies, subgroup analysis and sensitivity analysis were not carried out.

### 3.5 Relationship between the pretreatment PIV and DMFS

**Figure 5:**
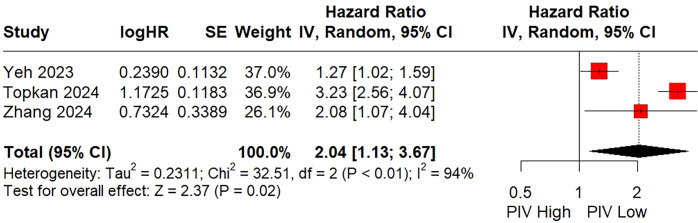
Forest plot assessing the relationship between the pretreatment PIV and distant metastasis-free survival.

There were three studies including 1,409 patients investigated the relationship between the PIV and DMFS using multivariate COX regression model. The random-effects pooled analysis showed that a higher PIV was significantly associated with a poorer DMFS (HR = 2.04; 95% CI: 1.13 - 3.67; P = 0.02; **Figure 5**). Although significant heterogeneity observed (I² = 94%), subgroup analysis and sensitivity analysis were not performed due to the limited number of included literature and consistent positive results among the included studies.

### 3.6 Publication bias

To assess potential publication bias related to primary outcomes, the funnel plot was used in combination with Begg’s and Egger’s tests. Concerning OS, the funnel plot showed asymmetry. Subsequent calculations gave Begg’s and Egger’s P values of 0.502 and 0.066 respectively, suggesting the existence of potential publication bias for this pooled outcome (**Supplementary Figure S4A**). Therefore, a trim-and-fill analysis was carried out, in which 5 additional potentially unpublished studies were incorporated. Nevertheless, the prognostic significance did not change (HR = 2.07; 95% CI: 1.51 – 2.85; P<0.01; I^2^ = 80%). Regarding DFS, the funnel plot was symmetric, with Begg’s and Egger’s P values of 0.602 and 0.222 respectively (**Supplementary Figure S4B**). For LRFS, the funnel plot was symmetric, with corresponding Begg’s and Egger’s P values of 0.296 and 0.196 respectively (**Supplementary Figure S4C**). As for DMFS, the funnel plot was symmetric, with Begg’s and Egger’s P values of 1.000 and 0.955 respectively (**Supplementary Figure S4D**).

## 4 Discussion

To the best of our knowledge, this study is the first systematic review and meta-analysis carried out to thoroughly explore the prognostic significance of the pretreatment PIV in HNSCC patients. For OS, pooled estimate derived from random-effects model demonstrated a significant association between elevated PIV and worse OS. Subgroup analyses further substantiated the potential of PIV as an independent prognostic biomarker across diverse clinical settings. Although the investigated variables did not explain the observed heterogeneity, sensitivity analysis indicated that the study by Lai et al. (25) was the primary source. Exclusion of this more prominent study did not alter the statistical significance of the pooled result. Moreover, despite detectable publication bias for OS, which may lead to overestimation, trim-and-fill analysis corroborated the robustness of the findings. For DFS, random-effects meta-analysis revealed a significant relationship between the PIV and DFS, with no evidence of publication bias. Subgroup analyses affirmed its prognostic relevance across different strata and identified sample size and tumor site as contributors to heterogeneity. Sensitivity analysis highlighted the study by Lien et al. (26) contributed to the significant heterogeneity. Excluding this more prominent study did not change the significance of the pooled result. In addition, our limited pooled data based on random-effects model also suggested that a higher PIV was related to worse DMFS and was marginally associated with poor LRFS in these patients.

The potential mechanism of PIV in predicting survival outcomes in HNSCC patients can be explained according to its specific components. Firstly, neutrophils, being the most prevalent innate immune cells, have been reported to assist tumor invasion and metastasis. Lonardi et al. (33) reported that a high frequency of CD66^+^ tumor-associated neutrophils (TANs) in the tumor-draining lymph nodes of oral squamous cell carcinoma patients correlates with poorer prognosis, suggesting that TANs may facilitate nodal dissemination via lymphatic vessels. Furthermore, elevated levels of PD-L1^+^ neutrophils have been shown to suppress T cell activation and promote immune evasion in tumor cells among HNSCC patients (34). Secondly, the abundance of monocytes, particularly those differentiating into tumor-associated macrophages (TAMs), is increased in HNSCC tissues compared with normal mucosa and is associated with unfavorable patient prognosis (35). Furthermore, TAMs exhibit multiple M2-like pro-tumoral properties, including promotion of tumor angiogenesis and extracellular matrix remodeling, which collectively facilitate tumor dissemination and confer resistance to radiotherapy in HNSCC patients (36, 37). Thirdly, platelets have been demonstrated to prompt tumor progression through the secretion of transforming growth factor-beta, vascular endothelial growth factor, and fibroblast growth factor in cancers (38). In oral squamous cell carcinoma, platelets can directly engage with podoplanin-expressing cancer cells through the C-type lectin-like receptor 2, thereby promoting cancer cell invasion and metastasis (39). Finally, lymphocytes serve as an important immune indicator and are crucial for immune surveillance and the defense against cancer (40). A decrease in lymphocyte levels in the tumor microenvironment can lead to an inferior prognosis in patients with HNSCC (41).

Recently, numerous studies have also reported the clinical value of the PIV in various malignancies. Cui et al. (42) reported that Wilms’ tumor Patients with higher PIV levels had significant poorer event-free survival, worse OS, and an increased likelihood of developing chemotherapy-related adverse events during treatment. A recent meta-analysis by Yu et al. (43) demonstrated that patients in the high PIV group exhibited significantly worse OS, DFS, PFS, recurrence-free survival, and cancer-specific survival when compared to those in the low PIV group. Yan et al. (44) demonstrated that PIV was as an independent predictor of the therapeutic effect in concurrent chemoradiotherapy for locally advanced cervical squamous cell carcinoma, and also an independent predictor of OS and DFS. Ucar et al. (13), investigating hematologic malignancies patients, revealed that a higher PIV before treatment correlated with poorer OS. Our meta-analysis results are consistent with these studies, providing further evidence supporting the promising potential of the PIV as an effective prognostic biomarker in patients with HNSCC.

Nevertheless, several limitations need to be recognized. First of all, all the included studies were retrospective in their design. This kind of design might bring about inherent selection bias and information bias that may affect the level of evidence. Secondly, the study population was composed solely of patients from China and Turkey. As a result, it is likely to restrict the general applicability of our research results to patients from other nations. Thirdly, the majority of patients included in this study initially underwent surgery or radiotherapy/chemoradiotherapy. Thus, additional investigations are necessary to establish the prognostic significance of the PIV in patients receiving neoadjuvant or systemic therapy. Lastly, a lack of a standardized PIV cut-off value across studies hindered its immediate clinical translation. Future studies may validate its prognostic value as a continuous variable.

## 5 Conclusions

Overall, the results of our pooled analysis indicate that the pretreatment PIV has the potential to be a valuable tool for prognostic evaluation in patients with HNSCC. Clinicians can make use of this informative indicator to categorize these patients and develop individualized treatment plans.

## Data Availability

The original contributions presented in the study are included in the article/**Supplementary Material**. Further inquiries can be directed to the corresponding author.
