# Supplementary material for: Prognostic value of the pretreatment pan-immune-inflammation value in patients with head and neck squamous cell carcinoma: a systematic review and meta-analysis
Source: Front Oncol. 2026 Mar 10;16:1743144. doi: 10.3389/fonc.2026.1743144 (PMC13008723; doi:10.3389/fonc.2026.1743144)
Supplement: Supplementary file 1 [file DataSheet1.docx]

| **Cohort study** | **Representatives of the exposed cohort** | **Selection of the non-exposed cohort** | **Ascertainment of exposure** | **Was outcome of interest present at start of study** | **Comparability of cohorts on the basis of the design or analysis** | **Assessment of outcome** | **Was follow-up long enough for outcomes to occur** | **Adequate follow up** | **Total** |
| --- | --- | --- | --- | --- | --- | --- | --- | --- | --- |
| Guven,2022 | 1 | 1 | 1 | 0 | 1 | 1 | 1 | 1 | 7 |
| Lai,2023 | 1 | 1 | 1 | 0 | 1 | 1 | 1 | 1 | 7 |
| Lien,2023 | 1 | 1 | 1 | 0 | 1 | 1 | 0 | 1 | 6 |
| Yeh,2023 | 1 | 1 | 1 | 0 | 1 | 1 | 1 | 1 | 7 |
| Koca,2024 | 1 | 1 | 1 | 0 | 1 | 1 | 1 | 1 | 7 |
| Shi,2024 | 1 | 1 | 1 | 0 | 1 | 1 | 0 | 1 | 6 |
| Shu,2024 | 1 | 1 | 1 | 0 | 1 | 1 | 1 | 1 | 7 |
| Topkan,2024 | 1 | 1 | 1 | 0 | 1 | 1 | 1 | 1 | 7 |
| Zhang,2024 | 1 | 1 | 1 | 0 | 1 | 1 | 1 | 1 | 7 |
| Chen (1),2025 | 1 | 1 | 1 | 0 | 1 | 1 | 1 | 1 | 7 |
| Chen (2),2025 | 1 | 1 | 1 | 0 | 1 | 1 | 1 | 1 | 7 |
| Huang,2025 | 1 | 1 | 1 | 0 | 1 | 1 | 1 | 1 | 7 |
| Li,2025 | 1 | 1 | 1 | 0 | 1 | 1 | 1 | 1 | 7 |

Table S1. Quality assessment of included studies using Newcastle-Ottawa Scale.


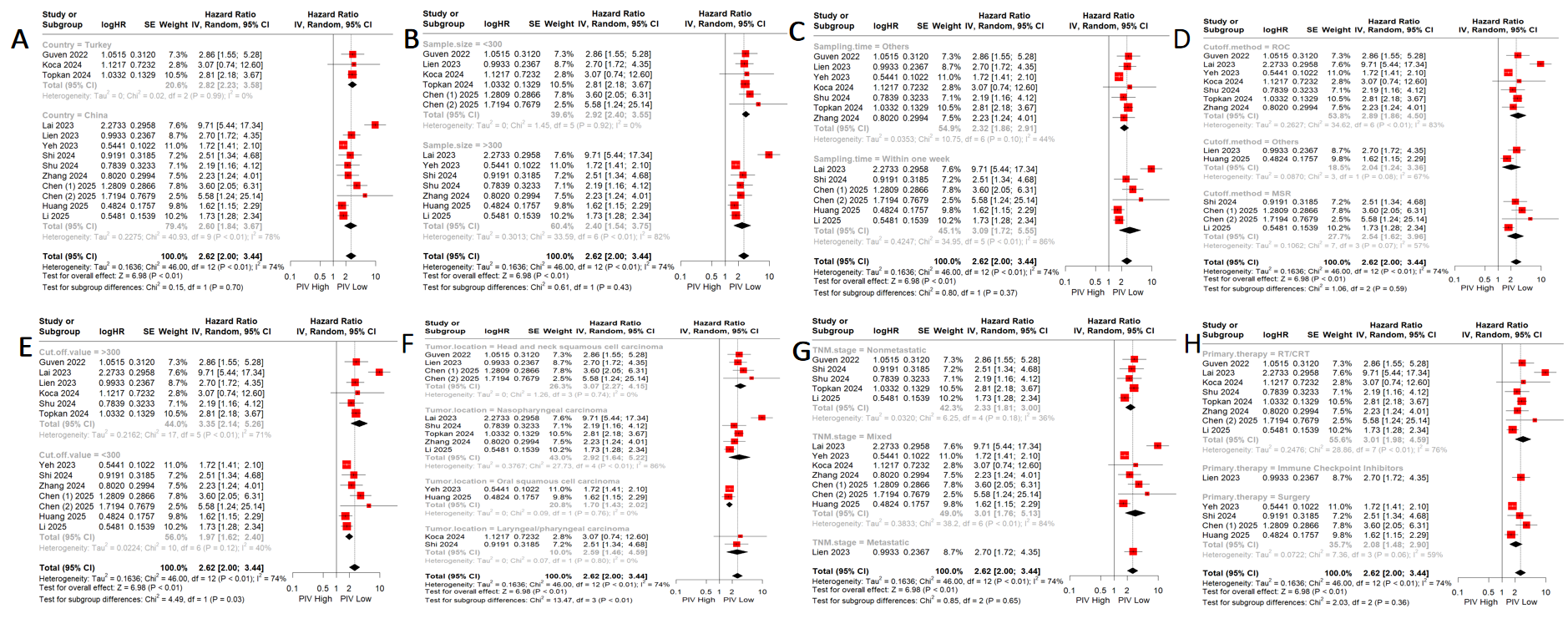


Figure S1. Forest plot of subgroup analyses assessing the relationship between the pretreatment PIV and OS. A: Country (China vs. Turkey); B: Sample size (>300 vs. <300); C: Sampling time (Within one week vs. Others); D: Cut-off method (ROC vs. MSR vs. Others); E: Cut-off value (>300 vs. <300); F: Cancer site (Head and neck squamous cell carcinoma vs. Nasopharyngeal carcinoma vs. Oral squamous cell carcinoma vs. Laryngeal/pharyngeal carcinoma); G: Tumor stage (Non-metastatic vs. Metastatic vs. Mixed); H: Primary treatment (Surgery vs. RT/CRT vs. ICI).

PIV: pan-immune-inflammation value; OS: overall survival; ROC: receiver operator characteristic; MSR: maximally selected rank; RT/CRT: radiotherapy or chemoradiotherapy; ICI: immune Checkpoint Inhibitor.


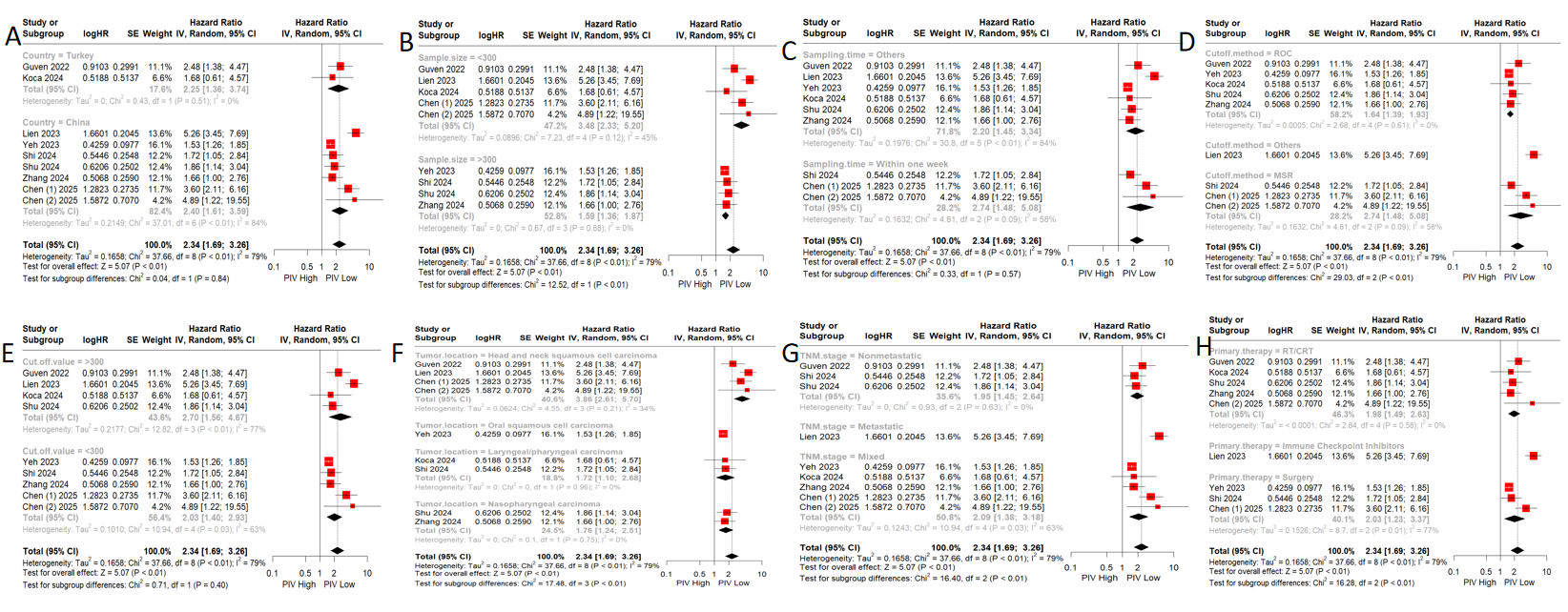


Figure S2. Forest plot of subgroup analyses assessing the relationship between the pretreatment PIV and DFS. A: Country (China vs. Turkey); B: Sample size (>300 vs. <300); C: Sampling time (Within one week vs. Others); D: Cut-off method (ROC vs. MSR vs. Others); E: Cut-off value (>300 vs. <300); F: Cancer site (Head and neck squamous cell carcinoma vs. Nasopharyngeal carcinoma vs. Oral squamous cell carcinoma vs. Laryngeal/pharyngeal carcinoma); G: Tumor stage (Non-metastatic vs. Metastatic vs. Mixed); H: Primary treatment (Surgery vs. RT/CRT vs. ICI).

PIV: pan-immune-inflammation value; OS: overall survival; ROC: receiver operator characteristic; MSR: maximally selected rank; RT/CRT: radiotherapy or chemoradiotherapy; ICI: immune Checkpoint Inhibitor.


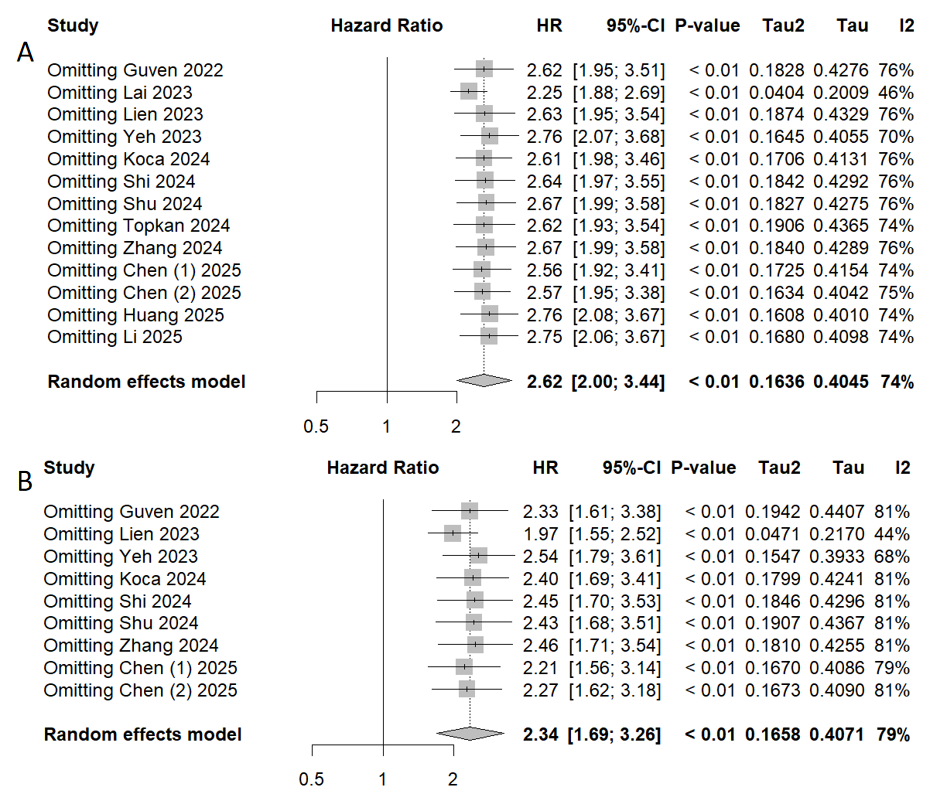


Figure S3. Sensitivity analyses assessing pooled outcomes, including (A) OS and (B) DFS.

OS: overall survival; DFS: disease-free survival.


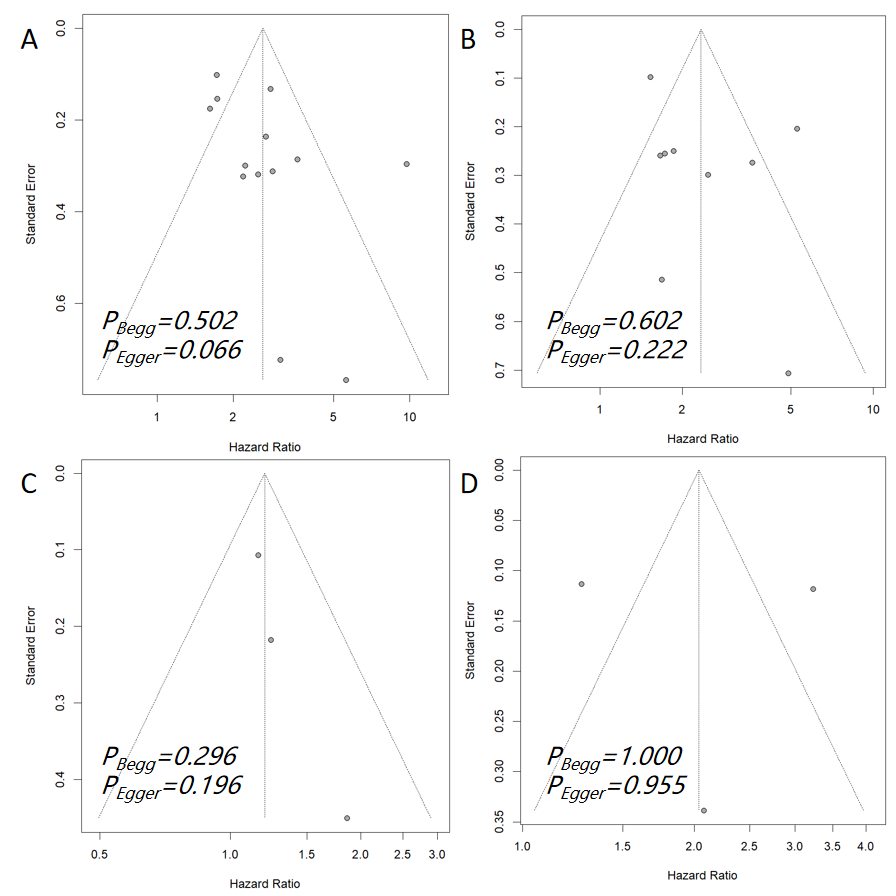


Figure S4. Funnel plots along with Eggers’ and Begg’s tests assessing publication bias between the pretreatment PIV and survival outcomes, including (A) OS, (B) DFS, (C) LRFS and (D) DMFS.

PIV: pan-immune-inflammation value; OS: overall survival; DFS: disease-free survival; LRFS: local recurrence-free survival; DMFS: distal metastasis-free survival.
